# Effects of Aggregate Size and Nozzle Diameter on Printability and Mechanical Properties of 3D Printed Ferronickel Slag–GGBFS Concrete

**DOI:** 10.3390/ma18153681

**Published:** 2025-08-05

**Authors:** Suguo Wang, Xing Wang, Xueyuan Yan, Shanghong Chen

**Affiliations:** College of Civil Engineering, Fuzhou University, Fuzhou 350108, China; wangsuguo@foxmail.com (S.W.); 18185976732@163.com (X.W.); chenshanghong@fzu.edu.cn (S.C.)

**Keywords:** aggregate size, printing nozzle, ferronickel slag and ground granulated blast-furnace slag (GGBFS), printability, mechanical properties

## Abstract

Ferronickel slag and ground granulated blast-furnace slag (GGBFS) are solid waste by-products from the metallurgical industry. When incorporated into concrete, they help promote resource utilization, reduce hydration heat, and lower both solid waste emissions and the carbon footprint. To facilitate the application of ferronickel slag–GGBFS concrete in 3D printing, this study examines how aggregate size and nozzle diameter affect its performance. The investigation involves in situ printing, rheological characterization, mechanical testing, and scanning electron microscopy (SEM) analysis. Results indicate that excessively large average aggregate size negatively impacts the smooth extrusion of concrete strips, resulting in a cross-sectional width that exceeds the preset dimension. Excessively small average aggregate size results in insufficient yield stress, leading to a narrow cross-section of the extruded strip that fails to meet printing specifications. The extrusion performance is closely related to both the average aggregate size and nozzle diameter, which can significantly influence the normal extrusion stability and print quality of 3D printed concrete strips. The thixotropic performance improves with an increase in the aggregate size. Both compressive and flexural strengths improve with increasing aggregate size but decrease with an increase in the printing nozzle size. Anisotropy in mechanical behavior decreases progressively as both parameters mentioned increase. By examining the cracks and pores at the interlayer interface, this study elucidates the influence mechanism of aggregate size as well as printing nozzle parameters on the mechanical properties of 3D printed ferronickel slag–GGBFS concrete. This study also recommends the following ranges. When the maximum aggregate size exceeds 50% of the nozzle diameter, smooth extrusion is not achievable. If it falls between 30% and 50%, extrusion is possible but shaping remains unstable. When it is below 30%, both stable extrusion and good shaping can be achieved.

## 1. Introduction

Against the backdrop of severe global climate change, reducing carbon emissions and achieving sustainable development have become a worldwide consensus. However, the world currently continues to face the challenge of high carbon emissions [1]. The construction industry, as one of the primary contributors, faces significant pressure to reduce emissions. An effective carbon reduction strategy involves the resource utilization of industrial solid wastes by incorporating them into concrete as partial replacements for cementitious materials, thereby reducing cement consumption and mitigating hydration reactions. Meanwhile, 3D printing technology (additive manufacturing), as an emerging construction method, reduces labor input and formwork usage during the printing process, which in turn lowers carbon emissions and construction waste generation [2]. Therefore, integrating 3D printing with solid waste blended concrete opens new avenues for low-carbon development in the construction sector.

Joseph Pegna [3] proposed a layer-by-layer construction method suitable for cementitious materials. Subsequently, Khoshnevis [4] first applied additive manufacturing technology to automated construction and employed contour crafting to print cement-based materials for building components. Recent studies have demonstrated that various solid wastes used as mineral admixtures in concrete exhibit distinct characteristics. When applied individually, these solid wastes have both advantages and limitations; however, their combined incorporation can generate synergistic effects that complement each other’s shortcomings. Le et al. [5] developed a high-performance concrete with remarkable workability by replacing cement with fly ash and silica fume, along with the addition of polypropylene fibers. Wang et al. [6] incorporated fly ash and silica fume additives to improve the pore structure within cement mortar and reduce anisotropy. Bong et al. [7] prepared 3D-printable alkali-activated materials using fly ash and slag. They investigated the effects of fly ash-to-slag mass ratios on the rheological properties and compressive strength of the alkali-activated matrix. Rahman et al. [8] investigated the trend of strength development in concrete with ferronickel slag as a single parameter, finding that concrete incorporating ferronickel slag exhibits relatively low early-age strength but experiences rapid strength development at later ages. Yin et al. [9] reported that the activity indices of ground blast furnace ferronickel slag and electric furnace ferronickel slag were 118% and 80%, respectively, with the poorest refined slag at only 73%, all meeting the requirements of Class II steel slag powder, thereby confirming their suitability as concrete additives for construction applications. It was also found that the incorporation of ferronickel slag leads to a decrease in the mechanical properties of concrete, while the addition of GGBFS enhances its mechanical performance. Tang [10] studied the influence of different replacement ratios of GGBFS powder and ferronickel slag powder as cementitious binders on the printability and mechanical properties of 3D printed concrete. It was also found that the incorporation of ferronickel slag improves the fluidity and slump of the concrete, which facilitates continuous extrusion, while the addition of ground GGBFS enhances the buildability of the concrete. Gao et al. [11] indicated that silica fume exhibits a spherical particle morphology, resembling tiny ball bearings, which can effectively reduce internal friction within the mixture. Its surface is smooth and dense, and its particle size is much smaller than that of cement and other admixtures. When used in combination with other mineral admixtures, silica fume can fully fill the pores in concrete, producing a dense packing effect that significantly enhances the compactness of the concrete structure. However, the current research on the 3D printing of concrete incorporating ferronickel slag and GGBFS is relatively limited in scope, and some parameters lack sufficient diversity. Therefore, further investigation and in-depth discussion are needed to clarify the effects of combined incorporation of ferronickel slag and GGBFS in concrete.

In 3D printed concrete, the proportion of coarse aggregates is relatively low. As a result, the cement content is over 25% higher than that in conventional concrete construction, leading to increased carbon emissions and material costs [12]. Addressing issues related to aggregate size in 3D printed concrete, Ding et al. [13] investigated the replacement of natural sand with recycled sand as aggregate in printable concrete. Although this approach reduced natural aggregate consumption, the high cement demand remained unresolved. An et al. [14] demonstrated that concrete containing coarse aggregates (with a maximum size of 4.75 mm, accounting for 30% of the total aggregate) offers advantages over conventional 3D printed concrete materials. These advantages include reduced shrinkage, lower cost, and decreased cement usage. Mechtcherine et al. [15] achieved 3D printing of coarse aggregate concrete with a maximum aggregate size of 8 mm using a small-scale printing device. Yuan et al. [16] developed a continuous and stable 3D-printing system capable of printing coarse aggregate concrete with a maximum aggregate size of 10 mm. Their comparative study showed that both compressive and flexural strengths exhibited anisotropic behavior. Microscopic observations further revealed a higher concentration of voids at the interlayer interfaces. Liu Huawei et al. [17,18] investigated the influence of pore defects in high coarse aggregate content 3D printed concrete on mechanical properties and proposed a “multi-zone interface model” to elucidate the underlying mechanism. Zhang et al. [19] studied the buildability and rheological properties of materials with different maximum aggregate size. Moreover, the properties of 3D printed concrete are significantly influenced by the nozzle diameter. Specimens printed with the same material but different nozzle sizes show notable differences, primarily in extrusion quality and the number of interlayer interfaces. At present, limited studies have addressed the effects of average aggregate size on 3D printed ferronickel slag–GGBFS concrete or explored the criteria for selecting the maximum aggregate size corresponding to different nozzle diameters.

**Figure 1:**
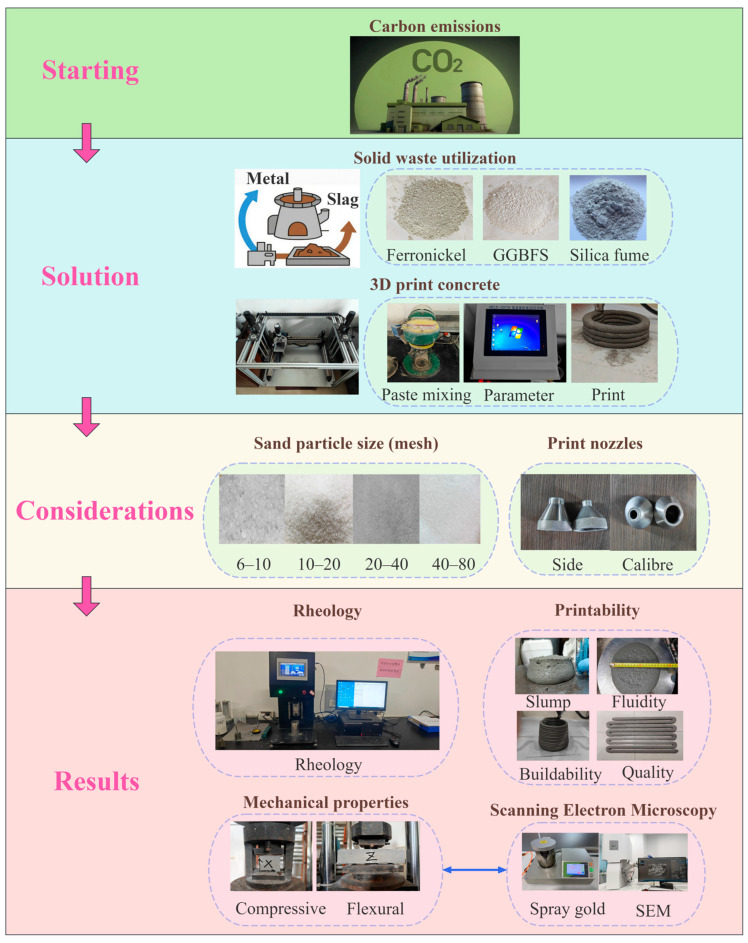
Research Background and Research Procedure.

In summary, both material factors (such as aggregate size, mineral admixtures, and chemical additives) and process parameters (including nozzle diameter, printing speed, and printing path) significantly influence the printability and mechanical properties of 3D printed concrete. Currently, further investigation is required on the effects of aggregate size and nozzle diameter, specifically, on 3D printed ferronickel slag–GGBFS concrete. Moreover, existing studies predominantly focus on single materials or individual parameters, lacking comprehensive evaluations of printability across various aggregate size–nozzle diameter combinations. Therefore, this study aims to assess the printability and mechanical properties of 3D printed ferronickel slag–GGBFS concrete by experimenting with five different aggregate size ratios and two nozzle diameters. The underlying mechanisms by which various factors influenced the performance variations were further analyzed and discussed. The research background and methodology are illustrated in Figure 1. Additionally, based on preliminary and formal experimental results, this paper proposes a preliminary recommended range of aggregate size–nozzle diameter combinations to achieve desirable printability performance.

## 2. Materials and Methods

### 2.1. Raw Materials

Ordinary Portland cement P·O42.5 (bagged) was used as the cement, with a 3-day compressive strength of 27.8 MPa and a 28-day compressive strength of 42.5 MPa, as well as a 3-day flexural strength of 5.2 MPa and a 28-day flexural strength of 6.5 MPa. Quartz sand was used as the fine aggregate, with particle size of 6–10 mesh (1.65–3.35 mm), 10–20 mesh (0.83–1.65 mm), 20–40 mesh (0.35–0.83 mm), and 40–80 mesh (0.2–0.35 mm). The specific surface areas of ferronickel slag and GGBFS were 415.6 m^2^/kg and 650.0 m^2^/kg, respectively. The silica fume used in this study was white in color, with a SiO_2_ content of 98%, a specific surface area of 19,100 m^2^/kg, and a spherical particle morphology. These supplementary cementitious materials were used to reduce the hydration heat of cement. A high-range water-reducing admixture (HRWR) and cellulose ether were added to enhance static yield stress and maintain fluidity. The water reducer exhibited a water-reduction rate of no less than 30%, while the cellulose ether had a fineness of 80–200 mesh and a viscosity of 200,000 mPa·s. In addition, redispersible polymer powder (RDP) with a particle fineness of approximately 80–120 mesh was incorporated to improve the adsorption between components within the concrete mixture. Furthermore, attapulgite clay with a specific surface area of 350.0 m^2^/kg was used as an adsorbent and catalyst carrier. The particles were dispersible, with diameters ranging from 20 to 40 nm and lengths between 1 and 5 μm.

### 2.2. Mix Proportion Design

The blending ratio of ferronickel slag and GGBFS was determined based on References [9,10] and preliminary experiments, which indicates that an equal-proportion mixture yields better printability and mechanical performance. Except for quartz sand, which was used as a variable, the proportions of all other components were kept constant. The detailed mix proportions of the concrete mortar are listed in Table 1. An orthogonal experimental design was adopted, with average aggregate size (particle gradation) and nozzle diameter as the variables. Five types of aggregate gradations were used, while the printing nozzle diameters were set to 10 mm and 20 mm. The specific aggregate gradation details are shown in Table 2.

Preliminary tests revealed that during the pre-experiment phase, aggregates with an aggregate size of 4–6 mesh (3.35–4.75 mm) could not be smoothly extruded through a 10-mm nozzle, accompanied by significant abnormal noises, indicating poor compatibility between the aggregate size and nozzle diameter. In contrast, when using a 20-mm nozzle, aggregates sized 4–6 mesh (3.35–4.75 mm) were extruded successfully. However, extrusion failure occurred when the maximum aggregate size reached 2–4 mesh (4.75–8 mm), which was consistent with the limitation observed for the 10-mm nozzle. Therefore, the maximum aggregate size for the 10-mm nozzle was maintained at 6–10 mesh (1.65–3.35 mm). The average aggregate particle diameter, denoted as *D* (Diameter), was calculated as follows in Equation (1):

(1) $$D=\alpha d_{1}+\beta d_{2}+\chi d_{3}+\delta d_{4}$$

The respective proportions of aggregate size are α, β, χ, δ;

$d_{1}$, $d_{2}$, $d_{3}$, $d_{4}$ denote the diameters of each aggregate size class, represented by their corresponding size ranges.

### 2.3. Experimental Methods for Evaluating Printability

The flowability test was performed in accordance with GB/T 2419-2005 [20] specification. Freshly mixed concrete was promptly poured into a standardized truncated cone mold, followed by tamping with a spatula and leveling the surface. The mold was then carefully lifted vertically to allow the mortar to spread freely. The filled mold was subjected to 25 jolts on a shaking table at a frequency of one shake per second. Subsequently, the average of the diameters measured along two perpendicular directions was recorded as the final flowability value.

For the rheological measurements, freshly mixed cement paste was immediately poured into a cylindrical container with an internal diameter of 60 mm and a height of 120 mm. The container was firmly fixed onto the testing platform to ensure stability during testing. A cross-shaped rotor with dimensions of 40 mm × 40 mm × 125 mm was inserted into the paste, and shear tests were conducted according to a predetermined shear rate protocol [21,22]. All measurements were carried out under controlled laboratory conditions to ensure accuracy and repeatability.

**Figure 2:**
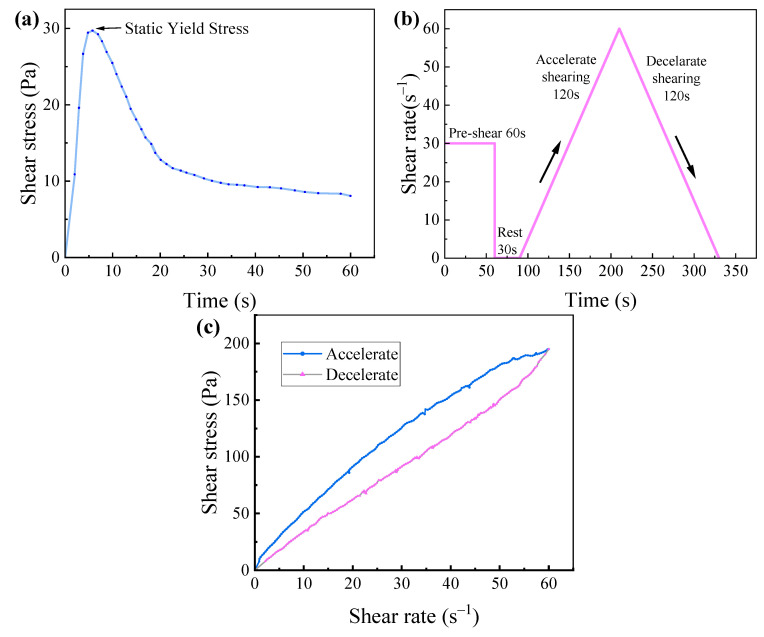
(**a**) Static shear procedure schematic (**b**) Dynamic shear procedure schematic (**c**) Thixotropy test procedure schematic.

Figure 2 presents schematic diagrams of the static shear procedure, dynamic shear test, and thixotropy test, respectively.

- Static Shear Procedure: The paste was sheared at a constant shear rate of 0.1 s^−1^ for 60 s. The peak shear stress obtained during this period was recorded as the static yield stress.
- Dynamic Yield Stress: A pre-shear at a shear rate of 30 s^−1^ was applied for 60 s to ensure uniform dispersion of the cement matrix, followed by a 30-s rest period. Subsequently, between 90 and 210 s, the shear rate was gradually increased from 0.1 s^−1^ to 60 s^−1^. During the interval from 210 to 330 s, the shear rate was uniformly decreased from 60 s^−1^ to 0 s^−1^, generating corresponding shear rate versus shear stress curves.
- Thixotropy Test: The thixotropic behavior of the 3D printed ferronickel slag–GGBFS concrete was characterized by the area enclosed between the rheological curves obtained during the increasing and decreasing shear rate segments, where the shear rate ranged from 0 to 60 s^−1^.

The buildability of 3D printed concrete was characterized by the number of stackable layers in a fixed structure and the height retention rate. The fixed structure was a hollow cylindrical specimen with a diameter of 100 mm and a height of 6 mm, formed by stacking 10 mm-wide strips. The average height was measured at four positions along two vertical directions. To minimize errors caused by standing time, buildability tests were conducted within 20 min after mixing with water. Extrudability refers to the ability of the cementitious paste to maintain suitable flowability, enabling uniform and continuous extrusion through the printing nozzle. It is typically evaluated by measuring the average cross-sectional width of the printed strips or the extrusion quality, which reflect the extrudability of the 3D printed concrete.

### 2.4. Methods for Mechanical Properties Test

The mechanical properties tests were conducted in accordance with GB/T 17671-1999 [23] specification. For compressive strength evaluation, 3D printed concrete specimens with dimensions of 40 mm × 40 mm × 40 mm were tested along the X, Y, and Z directions. A loading rate of 2.4 kN/s was applied, and the final compressive strength was obtained as the average value of six specimens. Flexural strength tests were conducted using 3D printed concrete specimens with dimensions of 40 mm × 40 mm × 160 mm, loaded along the Y and Z directions. A loading rate of 50 N/s was applied, and the final flexural strength was determined as the average value of three specimens.

**Figure 3:**
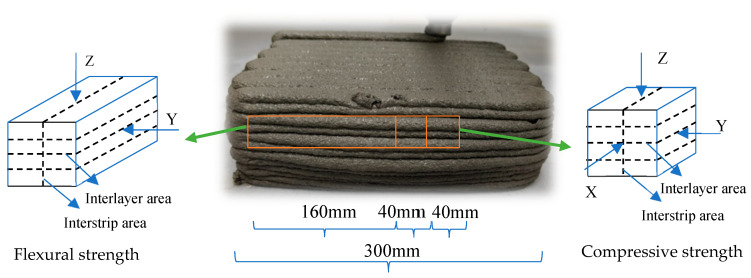
Schematic diagram of loading directions for compressive and flexural strength tests.

The loading configurations for both compressive and flexural strength tests were illustrated in Figure 3.

## 3. Results and Analysis of Printing Tests

### 3.1. Analysis of Printability

The study investigated the printability of ferronickel slag–GGBFS concrete under different aggregate size. Table 3 presented the flowability, slump, extrusion quality, cross-sectional width, and number of printable layers for mix groups SD-1 to SD-5.

#### 3.1.1. Flowability and Slump

Flowability and slump tests were conducted on concrete mixtures with different aggregate sizes from mix groups SD-1 to SD-5. The results indicated that aggregate size had a significant influence on both flowability and slump. As the average aggregate size increased, the flowability of the 3D printed ferronickel slag–GGBFS concrete exhibited a clear upward trend, while the slump showed a decreasing trend. All SD-1 to SD-5 mixes demonstrated uniform and continuous extrusion behavior during printing. The measured flowability ranged from 169 mm to 185 mm, and the slump ranged from 32 mm to 58 mm.

#### 3.1.2. Extrudability

According to the flowability analysis, all mix groups from SD-1 to SD-5 exhibited continuous extrusion behavior during the printing process. With the increase in average aggregate size, both the cross-sectional width of the printed strips and the extrusion quality improved. This phenomenon is primarily attributed to the decrease in particle fineness, which results in a reduction in particle packing density and a decrease in paste flowability. Consequently, the yield stress of the material increases, leading to reduced extrusion quality and narrower strip cross-sections [24].

#### 3.1.3. Buildability

Under the condition of satisfactory extrudability, buildability tests were conducted on mix groups SD-1 to SD-5 to further investigate the influence of aggregate size on the buildability of ferronickel slag–GGBFS concrete. All five groups successfully achieved a stackable height of 12 layers, primarily attributed to the activation of GGBFS, which promotes the pozzolanic reaction with calcium hydroxide to generate secondary C–S–H gels (along with a minor formation of C–A–S–H) [25]. This reaction enhances interlayer bonding strength and early-age structural stability, thereby improving the overall buildability and interfacial adhesion of the printed layers. However, due to the smaller average aggregate size, the SD-1 mix exhibited relatively lower yield stress and plastic viscosity, making it more prone to excessive deformation during extrusion. As a result, when extruded with 10-mm and 20-mm nozzles, the cross-section of the extruded strips in the SD-1 group was smaller than the nozzle diameter, with slight inward deformation of the printed layers occurring around the 10th to 12th layers when using the 10-mm nozzle. In contrast, the other groups demonstrated better height retention and build stability.

Compared with the synergistic effect of fly ash and slag, fly ash—with its spherical particle shape—helps improve the flowability of the paste during 3D printing and reduces the tendency for collapse or deformation after extrusion. In contrast, slag contributes to the overall structural integrity of the mixture due to its high latent hydraulic activity and filling capacity [26]. Ferronickel slag, on the other hand, consists of angular, irregular particles with a dense structure and relatively smooth surfaces. These characteristics may introduce uneven pore structures, potentially affecting the homogeneity and printing stability of the mix. To mitigate this adverse effect, silica fume was incorporated in this study as a supplementary cementitious material to refine the microstructure, fill voids, and enhance the continuity and bonding performance of the mixture.

Increasing aggregate size can improve mixture printability to some extent. However, when the maximum aggregate size exceeds a critical threshold, the nozzle fails to extrude the concrete strips properly. Printing experiments confirmed that when the maximum aggregate size is approximately 50% of the nozzle diameter, extrusion cannot be achieved. When the size ranges between 30% and 50% of the nozzle diameter, concrete can be extruded, but the strips are poorly formed and discontinuous. In contrast, when the maximum aggregate size is below 30% of the nozzle diameter, the strips can be extruded smoothly, and printability is optimal when the maximum aggregate size approaches 30% of the nozzle diameter.

### 3.2. Rheological Performance Analysis

#### 3.2.1. Static Yield Stress

**Figure 4:**
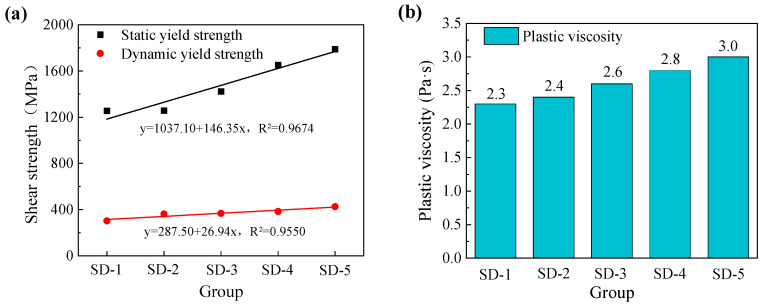
(**a**) Static and dynamic yield stress of 3D printed ferronickel slag–GGBFS concrete (**b**) Plastic viscosity of 3D printed ferronickel slag–GGBFS concrete.

The initial static yield stress of mortar mixtures with five different aggregate size proportions was investigated. The shear stress behavior of 3D printed ferronickel slag–GGBFS concrete under a constant shear rate exhibited a similar trend across all mixtures: an initial increase to a peak value, followed by a gradual decrease and eventual stabilization. Figure 4a shows the fitted curve between aggregate size proportion and static yield stress, revealing a strong positive linear correlation with a coefficient of determination R^2^ = 0.9550. This indicates that the aggregate size has a linear relationship with the static yield stress of ferronickel slag–GGBFS concrete, which macroscopically corresponds to an enhanced resistance to deformation. Therefore, appropriately increasing the aggregate size can improve the extrudability of the ferronickel slag–GGBFS concrete.

#### 3.2.2. Dynamic Yield Stress

Dynamic yield stress refers to the minimum shear stress required to maintain the material in a flowing state. When the applied shear stress exceeds the critical yield stress, the material’s internal structure breaks down, causing the paste to flow. Plastic viscosity represents the difficulty of disrupting the colloidal system under shear, reflecting the magnitude of the opposing viscous resistance relative to the flow direction among the flow layers of the paste. The Bingham model, suitable for non-Newtonian materials exhibiting yield stress [27], was employed to fit the shear stress versus shear rate data in the decreasing shear rate range from 50 to 10 s^−1^. The intercept of the fitted curve corresponds to the dynamic yield stress, while the slope represents the plastic viscosity. Figure 4 indicates that with increasing aggregate size, both the dynamic yield stress and plastic viscosity of the 3D printed ferronickel slag–GGBFS concrete increase. The fitted curves for different aggregate size proportions exhibit a strong correlation with a coefficient of determination R^2^ = 0.9674.

#### 3.2.3. Thixotropy

The formation of the hysteresis loop is primarily attributed to the fact that, under a constant shear rate, the shear stress during the acceleration phase of the paste is higher than that during the deceleration phase. During acceleration, the paste endures elevated shear stress to break down the flocculated structure formed by cement hydration products and the intermolecular van der Waals forces. At this stage, the rate of structural breakdown exceeds the rate of rebuilding, resulting in a decrease in apparent viscosity. Conversely, during the deceleration phase, the structural rebuilding dominates as the flocculated structures and interaction forces gradually recover, causing the apparent viscosity to increase.

The hysteresis loop area is used to characterize the thixotropic behavior of the mortar, simulating the entire process from internal rotation within the printer to extrusion.

**Figure 5:**
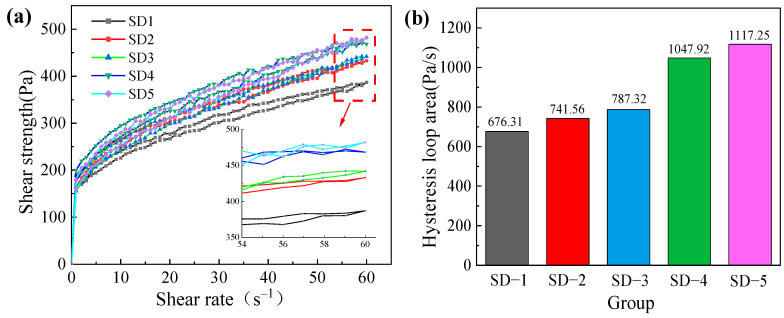
(**a**) Thixotropic hysteresis loop (**b**) Hysteresis loop area of mortar in the initial state.

As shown in Figure 5, the hysteresis loop area increases progressively with the average aggregate size. Specifically, for the aggregate size proportions in groups SD-1 through SD-5, the corresponding hysteresis loop areas are 676.31 Pa/s, 741.56 Pa/s, 787.32 Pa/s, 1047.92 Pa/s, and 1117.25 Pa/s, respectively. Among all mixtures, the SD-5 group, containing the highest proportion of large particles, exhibits the most pronounced thixotropic behavior. In comparison, the hysteresis loop areas of SD-1, SD-2, SD-3, and SD-4 decrease by 39.47%, 33.63%, 29.53%, and 6.21%, respectively, relative to SD-5. Notably, in mixtures SD-4 and SD-5, which include aggregate particles of 1.65–3.35 mm in diameter, the hysteresis loop area rapidly increased and gradually leveled off. This phenomenon is attributed to greater fluctuations in the paste induced by the larger particles during rotor rotation.

## 4. Mechanical Properties Test Results and Analysis

### 4.1. Compressive Strength Test Results and Analysis

**Figure 6:**
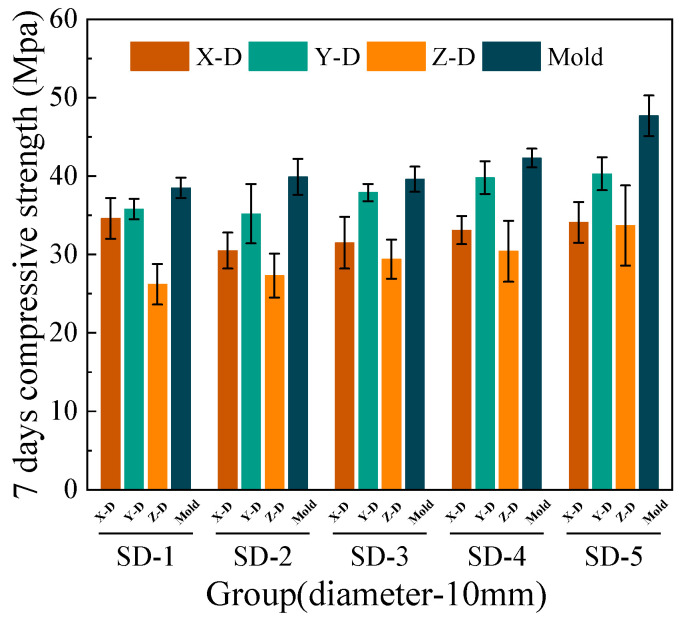
7-day compressive strength (10-mm nozzle).

**Figure 7:**
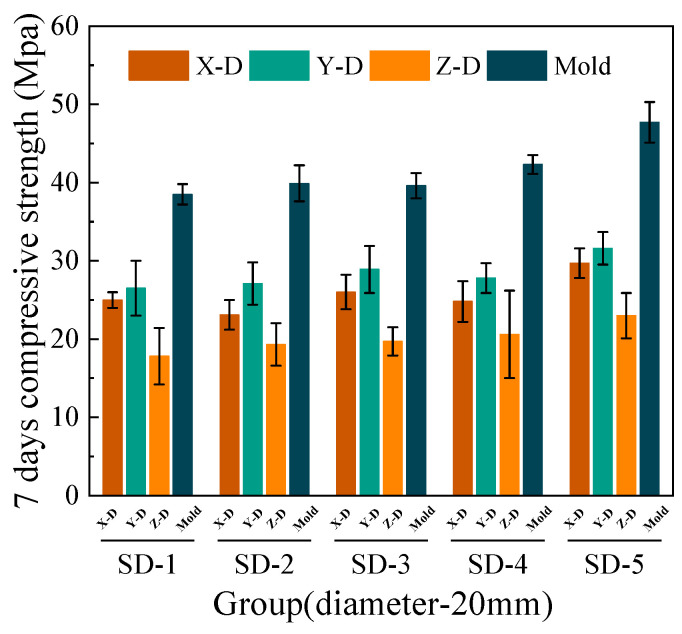
7-day compressive strength (20-mm nozzle).

**Figure 8:**
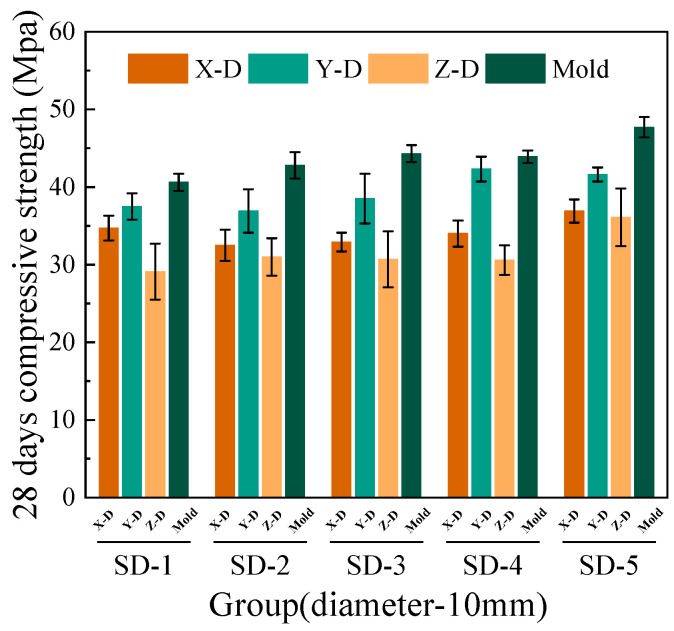
28-day compressive strength (10-mm nozzle).

**Figure 9:**
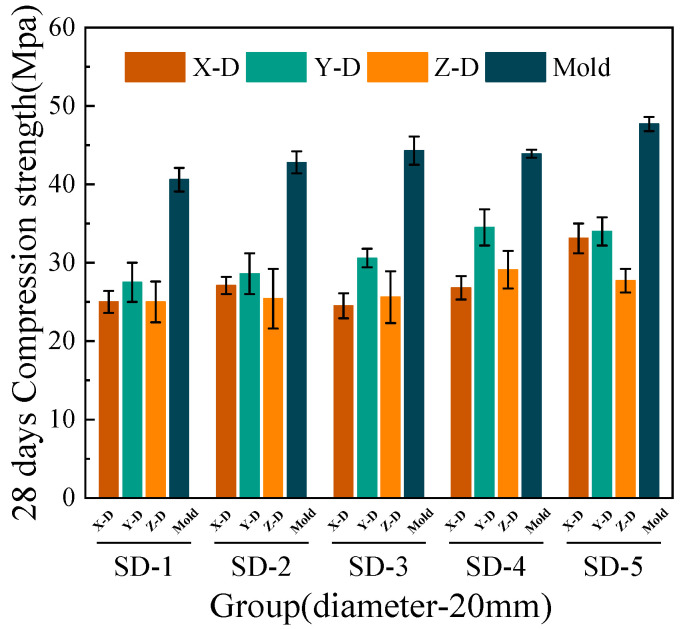
28-day compressive strength (20-mm nozzle).

Figure 6, Figure 7, Figure 8 and Figure 9 illustrate that under mix proportions SD-1 to SD-5, the compressive strength increases with the average aggregate size. A control group of conventionally cast concrete was prepared by pouring the mixture into molds, referred to as “Mold” in the figure for simplicity. Additionally, a noticeable difference exists between the compressive strengths of 3D printed ferronickel slag–GGBFS concrete and conventionally cast specimens. The results indicate that 3D printed specimens fabricated with a 10-mm nozzle generally exhibit higher compressive strength than those printed with a 20-mm nozzle. This is primarily attributed to insufficient extrusion pressure during printing; when using a larger nozzle diameter, the extruded paste tends to be more loosely packed, resulting in increased internal porosity (detailed analysis is presented in Section 4 on microstructure).

In conventional concrete, mechanical properties typically improve with increasing aggregate size; however, beyond a certain threshold, the internal compactness may decrease, causing strength degradation [28]. Conversely, in concrete incorporating ferronickel slag and GGBFS, mechanical properties remain stable or even continue to improve within the printable aggregate size range. This is primarily attributed to the pozzolanic reactivity of GGBFS, which promotes the formation of additional cementitious products (e.g., C–S–H gel) between the cement paste and aggregates. As a result, the interfacial transition zone (ITZ) becomes denser and exhibits improved bonding performance. The enhanced interfacial cohesion reduces weak planes between printed layers. This suggests that the industrial solid waste blended system exhibits lower sensitivity to aggregate size variations and demonstrates enhanced interfacial compatibility.

The 7-day and 28-day compressive strengths of specimens printed with 10-mm and 20-mm nozzles are lower than those of cast concrete, with the specific reduction levels detailed in Table 4. Furthermore, a comparison of compressive strengths along the X, Y, and Z directions reveals that the Y direction exhibits the highest strength, followed by the X direction, while the Z direction has the lowest strength. This phenomenon is mainly attributed to the fact that, during printing, the cross-sectional width of the extruded strips exceeds the nozzle diameter. The strips are laterally compressed by adjacent strips, resulting in better compaction along the Y direction and thus enhancing compressive strength in this direction [29].

Under the condition of a 20-mm nozzle diameter, the reduction in compressive strength at 28 days relative to 7 days is smaller across all mix proportions, indicating a relatively slower early strength development compared to cast concrete, followed by a rapid strength gain at later ages. In contrast, for the 10-mm nozzle condition, the decrease in compressive strength between 7 and 28 days is more consistent across all mixes, suggesting that the early and late compressive strength development trends align closely with those of cast concrete. The findings differ slightly from those of M.A. et al. [26], which proposed ferronickel slag blended into cast concrete exhibits slow early-age mechanical properties development followed by rapid strength gain at later ages, achieving strength comparable to that of concrete without supplementary cementitious materials. These findings indicating that variations in aggregate size and nozzle diameter have limited impact on the compressive strength development of ferronickel slag–GGBFS concrete. When ferronickel slag and GGBFS are incorporated into concrete as blended binders, a reduction in early-age strength is observed. However, as cement hydration progresses, the later-age strength of the concrete improves significantly, primarily because silica fume and ferronickel slag addition enhance early strength development.

### 4.2. Flexural Strength Test Results and Analysis

**Figure 10:**
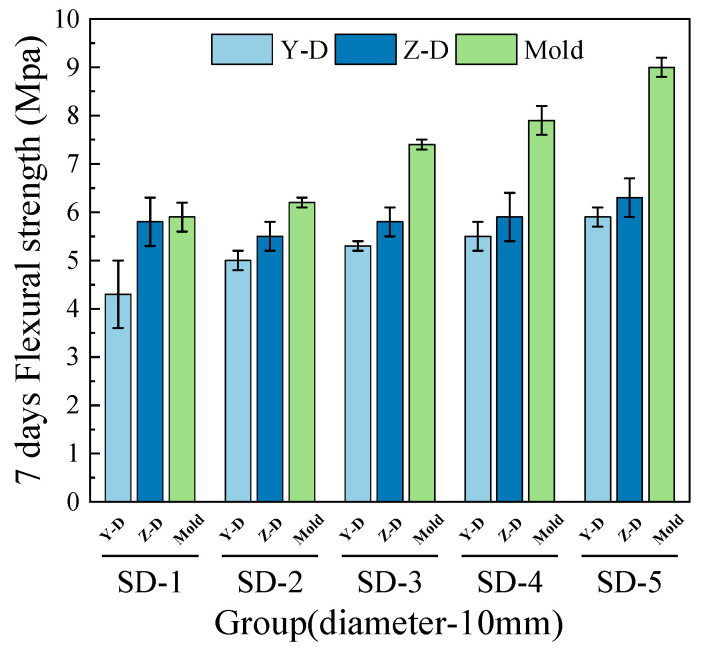
7-day flexural strength (10-mm nozzle).

**Figure 11:**
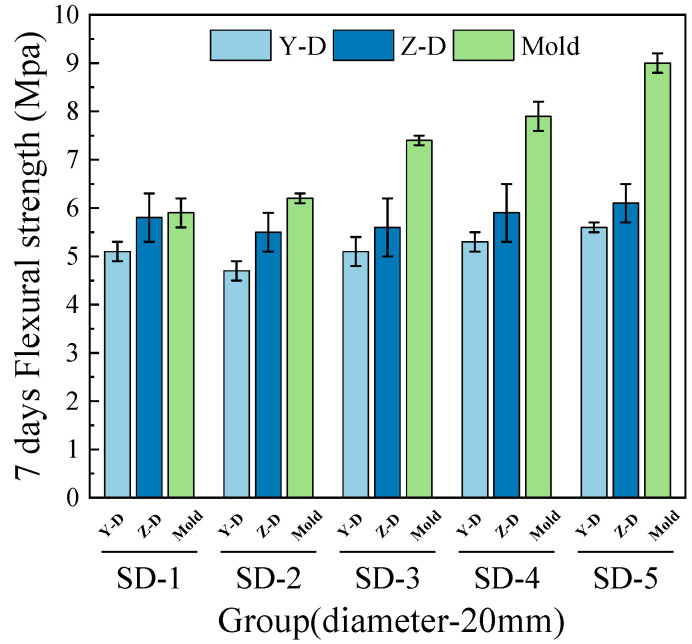
7-day flexural strength (20-mm nozzle).

**Figure 12:**
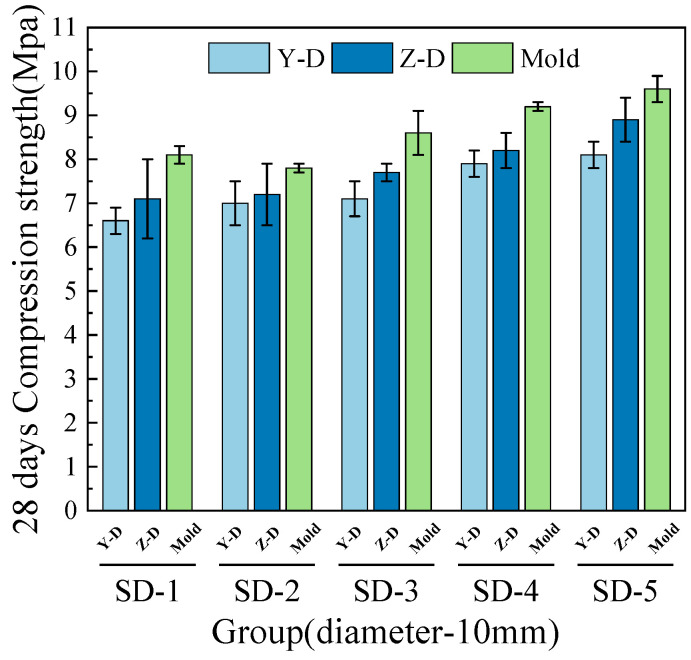
28-day flexural strength (10-mm nozzle).

**Figure 13:**
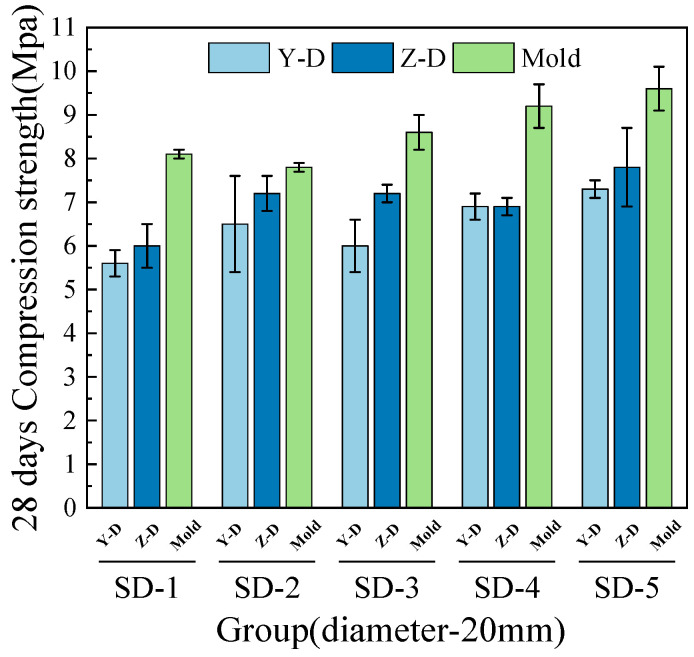
28-day flexural strength (20-mm nozzle).

As shown in Figure 10, Figure 11, Figure 12 and Figure 13, the flexural strength of specimens from mix groups SD-1 to SD-5 increases with the average aggregate size. The overall flexural strength of the 3D printed ferronickel slag–GGBFS concrete with a 10-mm nozzle is higher than that obtained using a 20-mm nozzle. Additionally, the results reveal noticeable differences in flexural strength between the 3D printed specimens and the cast specimens molded using conventional methods.

In addition, a comparison of flexural strength in the Y and Z directions shows that the Z-direction strength is slightly higher than that of the Y direction. Compared to cast specimens, the average flexural strength of all 3D printed specimens decrease. Both the 7-day and 28-day flexural strengths of specimens printed with 10-mm and 20-mm nozzles are lower than those of cast concrete, with specific reduction values detailed in Table 4.

Under the 20-mm nozzle condition, the reduction in flexural strength at 28 days for all mix proportions was relatively small, indicating a slower early-age development and a subsequent increase in flexural strength at later ages. The results are consistent with the findings of Rahman. et al. [8] on cast concrete containing ferronickel slag, indicating that ferronickel slag exhibits weak reactivity at early ages, but its activity is gradually activated with the progression of hydration, thereby promoting the development of later-age strength. This also suggests that variations in aggregate size and nozzle diameter have limited influence on the flexural strength development of ferronickel slag–GGBFS concrete.

### 4.3. Anisotropy Analysis

There is a significant difference in the mechanical properties between 3D printed concrete and traditionally cast concrete. For cast concrete, specimens are molded with formwork and compacted by vibration, resulting in relatively uniform properties in all directions, and thus can generally be regarded as isotropic materials. However, due to the influence of multiple factors during the printing process, the cross-sectional dimensions and geometrical shape of 3D printed concrete components are often difficult to control precisely during forming, which affects their mechanical properties to a certain extent [30].

Therefore, to better analyze the anisotropy of the printed specimens, an anisotropy coefficient (*I_a_*) is introduced to characterize the directional behavior of 3D printed concrete. Currently, researchers both in China and abroad have proposed various definitions for the anisotropy coefficient. In this study, the method proposed by Wang et al. [31] is adopted for calculating the anisotropy coefficient, as shown in Equations (2) and (3).

(2) $$I_{a}=\sqrt{(f_{cx}-f_{cavg})+{\left(f_{cy}-f_{cavg}\right)}^{2}+{\left(f_{cz}-f_{cavg}\right)}^{2}}$$

In the equation, $I_{a}$—represents the anisotropy coefficient;

$f_{cx}$, fcy, $f_{cz}$—3D printed concrete compressive strength in the X, Y, and Z directions, MPa;

$f_{cavg}$—average compressive strength of 3D printed concrete in the X, Y, and Z directions, MPa.

(3) $$I_{a}=\sqrt{{\left(f_{ly}-f_{lavg}\right)}^{2}+{\left(f_{lz}-f_{lavg}\right)}^{2}}$$

$f_{ly}$, $f_{lz}$—3D printed concrete flexural strength in the Y, and Z directions, MPa;

$f_{lavg}$—average flexural strength of 3D printed concrete in the Y, and Z directions, MPa.

As shown in Table 5, the anisotropy coefficients (*I_a_*) of the compressive and flexural strengths of 3D printed concrete vary with different curing ages, aggregate size, and printing nozzle diameters. The 7-day compressive strength anisotropy coefficients range from 5.11 to 7.40, decreasing to 2.04–6.05 at 28 days, demonstrating significant anisotropy. In contrast, the 7-day flexural strength anisotropy coefficients range from 0.28 to 1.06 and decrease to 0–0.92 at 28 days, indicating relatively low anisotropy. Variations in aggregate size and nozzle diameter minimally affect the flexural strength anisotropy of 3D printed ferronickel slag–GGBFS concrete. The incorporation of ground granulated blast-furnace slag (GGBFS) enhances the pozzolanic reactivity of the mixture, promoting the formation of additional cementitious products, such as calcium silicate hydrate (C–S–H) gels, at the interface between the cement paste and aggregates. This contributes to a denser and more cohesive interfacial transition zone (ITZ), which significantly improves the interlayer bonding. As a result, the presence of weak interfaces between printed layers is reduced, thereby decreasing performance discrepancies along the printing direction and mitigating the anisotropy typically observed in 3D printed concrete. These results demonstrate that 3D printed ferronickel slag–GGBFS concrete shows good applicability. Moreover, enhancing its mechanical properties through the optimization of aggregate size and the selection of appropriate nozzle diameters is considered highly feasible.

## 5. Microstructure Morphology and Analysis

The interlayer microstructure in the Z-direction was observed using scanning electron microscopy (SEM) to further evaluate the structural variations caused by different printing nozzle diameters and aggregate size, and to elucidate the underlying mechanisms affecting the mechanical properties.

### 5.1. Microstructure of Interlayer Interfaces Under Different Nozzle Diameters

**Figure 14:**
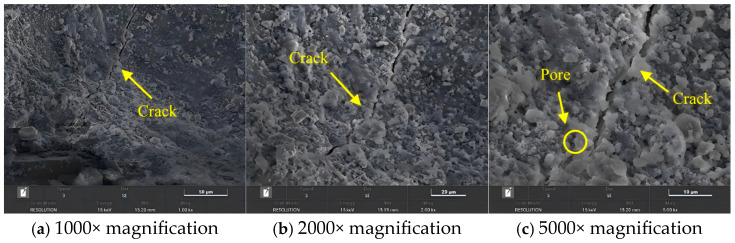
Cracks and aggregate size at the interlayer interface under different magnifications for SD-5 mix (10-mm nozzle).

**Figure 15:**
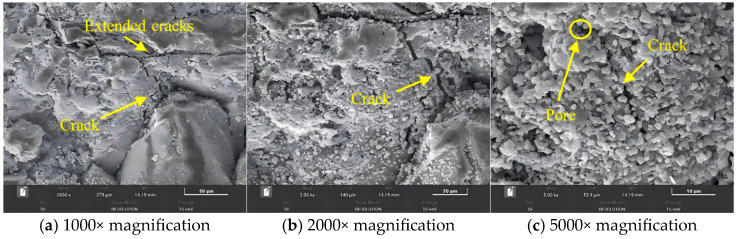
Cracks and aggregate size at the interlayer interface under different magnifications for SD-5 mix (20-mm nozzle).

Taking the SD-5 mix as an example, printing was performed using 10-mm and 20-mm nozzles, respectively, to observe the microstructural variations of the Z-direction interlayer bands. As shown in Figure 14 and Figure 15, at three different magnifications, the interlayer interface cracks are wider when printed with the 20-mm nozzle, accompanied by a greater number of pores around the cracks. In contrast, samples printed with the 10-mm nozzle exhibited narrower cracks and fewer surrounding pores. Combined with mechanical test results, it is concluded that the primary cause of strength reduction in components printed with larger nozzles is the more pronounced interlayer cracking and decreased compactness of the pore structure in the crack regions. After incorporating ferronickel slag and GGBFS industrial wastes, more micro-pores are formed inside the carbonated concrete. Although the size of individual pores decreases, the total number of pores significantly increases [32], leading to an overall more porous structure, which is one of the main reasons for the reduction in concrete strength.

Additionally, as observed in Figure 14, distinct interlayer interface zones were formed during the 3D printing process, where the distribution of hydration products was relatively sparse and the interlayer bonding is limited. This phenomenon is one of the key factors contributing to the anisotropic behavior of 3D printed concrete. In contrast, cast concrete specimens exhibited a dense structure, whereas the interfacial zones in the microstructure of 3D printed specimens contained more interconnected pores, resulting in a comparatively looser structure [33].

### 5.2. Microstructure of Interlayer Interfaces with Different Aggregate Size Incorporated

**Figure 16:**
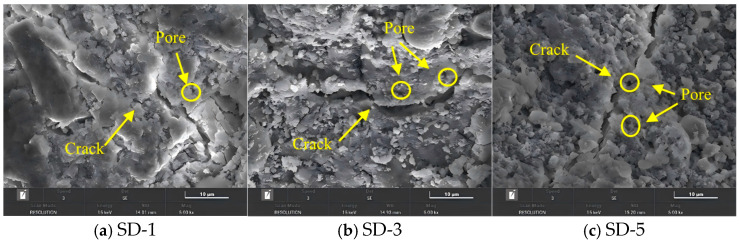
Distribution of different aggregate sizes (10-mm nozzle, 5000× magnification).

**Figure 17:**
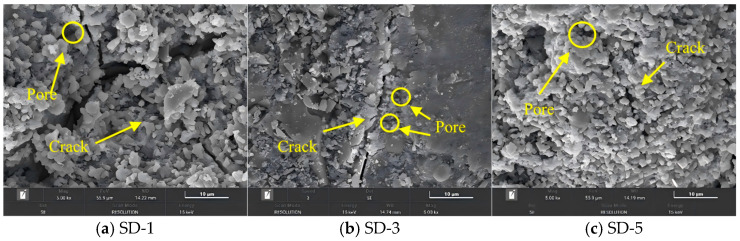
Distribution of different aggregate sizes (20-mm nozzle, 5000× magnification).

As shown in Figure 16 and Figure 17, under the appropriate aggregate gradation, the interlayer crack widths for SD-1, SD-3, and SD-5 (with 10-mm and 20-mm nozzle diameters) gradually decreased, indicating an increase in the internal compactness of the concrete structure. Meanwhile, when coarse aggregate is used in 3D printed ferronickel slag–GGBFS concrete, the large particles can penetrate interlayer cracks and thereby enhance the interfacial bonding between printed layers. This variation explained the observed improvement in mechanical properties with increasing average aggregate size. Therefore, when designing mix proportions for 3D printed ferronickel slag–GGBFS concrete, a larger average aggregate size should be selected to ensure good printability while enhancing mechanical properties. However, the aggregate size distribution must comply with the packing principle to avoid insufficient aggregate interlock, which could lead to a loose internal structure and negatively affect overall properties. When larger aggregate sizes are used, the total surface area of aggregates per unit volume decreases, allowing the paste to be more concentrated within the voids between aggregates. This contributes to the development of a more homogeneous and compact microstructure, characterized by a continuous distribution of C–S–H gel and reduced porosity. In contrast, smaller aggregate sizes result in a greater total surface area, causing the paste to be more dispersed and potentially increasing the local water-to-binder ratio. This may lead to the formation of more pores or weakly hydrated regions, thereby affecting the quality and density of C–S–H gel formation.

Notably, when coarse aggregates with larger aggregate sizes are used, the aggregate particles tend to span across the interlayer interfaces, forming a “bridging effect.” This effect can effectively reduce the width of interfacial cracks and suppress their further propagation, while also enhancing the mechanical interlocking between adjacent layers, thereby improving overall structural integrity. In contrast, smaller aggregates are generally confined within individual layers and have limited ability to enhance interlayer bonding.

Meanwhile, the weak interlayer zones also explain why the mechanical properties of 3D printed concrete specimens are inferior to that of cast specimens and the existence of anisotropy. In studies investigating the influence of aggregate size on 3D printed ferronickel slag–GGBFS concrete, the cement content used in the 3D printed ferronickel slag–GGBFS concrete was reduced by 16.2–20.0% compared to previous research on 3D printed mortars [10,34,35], thereby reducing the hydration heat generated by the hydration reaction, which in turn contributed to a partial reduction in carbon emissions.

## 6. Conclusions

Through systematic analysis and research on the printing performance, mechanical properties, and interlayer interface microstructure of 3D printed ferronickel slag–GGBFS concrete under varying aggregate size and printing nozzle diameters, the following conclusions have been drawn:

(1) Ferronickel slag and GGBFS can be used as supplementary cementitious materials in concrete, partially replacing cement to reduce its consumption and lower carbon emissions. Experimental data demonstrate that the incorporation of blended solid wastes, due to their synergistic effect, significantly enhances the printability and mechanical properties of concrete, ensuring the successful and continuous extrusion of concrete strips.

(2) The aggregate size significantly affects the extrudability of 3D printed ferronickel slag–GGBFS concrete. When the average aggregate size is too large, the extrusion smoothness of concrete strips is impaired, and the extruded strip cross-section is usually larger than the preset width; conversely, too small of an aggregate size led to undersized cross-sections that do not meet printing requirements. This indicates that extrudability correlates with nozzle diameter and average aggregate size. When the maximum aggregate size exceeds 50% of the nozzle diameter, extrusion fails. Within 30–50%, extrusion is possible but results in poorly formed strips. Optimal and continuous printing occurs when the maximum aggregate size is below 30%, especially near the 30% threshold.

(3) As the aggregate size increases, the compressive and flexural strengths of the 3D printed ferronickel slag–GGBFS concrete show an increasing trend, indicating that larger aggregate size contributes to improved overall structural load-bearing capacity. Larger aggregate size also reduces the width of interfacial cracks and enhances the internal density of the concrete. Changes in aggregate size do not significantly affect the anisotropy of compressive strength, while anisotropy in flexural strength is less pronounced. Ferronickel-slag blended concrete exhibits better stability and tolerance in mechanical properties, indicating higher adaptability to aggregate size, which helps improve strength and broaden aggregate selection.

(4) Increasing the nozzle diameter results in wider interlayer interface cracks and more continuous pores around the cracks, leading to a more porous overall structure and, thus, reduced mechanical properties. The variation in nozzle diameter has little effect on the anisotropy of the 3D printed ferronickel slag–GGBFS concrete itself; the compressive strength still exhibits significant anisotropy, while the flexural strength remains largely isotropic.

## Figures and Tables

**Table 1:** 3D Printed concrete mix ratio.

Quartz Sand	P·O42.5	Ferro Nickel	GGBFS	Silica Fume	Water	Water Reducer	Cellulose Ether	Attapulgite Clay	RDP
1500 g	600 g	150 g	150 g	100 g	352 g	0.14%	0.15%	0.3%	0.08%

**Table 2:** Allocation of experimental groups and variables.

Group	6–10 Mesh (1.65–3.35 mm)	10–20 Mesh (0.83–1.65 mm)	20–40 Mesh (0.35–0.83 mm)	40–80 Mesh (0.2–0.35 mm)	Average Diameter (mm)
SD-1	0	20%	40%	40%	0.39–0.80
SD-2	0	40%	20%	40%	0.48–0.97
SD-3	0	40%	40%	20%	0.51–1.06
SD-4	20%	30%	30%	20%	0.72–1.48
SD-5	40%	20%	20%	20%	0.94–1.91

**Table 3:** Printability evaluation of each group.

Group	Extruded Quality (g)	Width-10 mm (mm)	Width-20 mm (mm)	Strips (10 mm)	Fluidity (mm)	Slump (mm)
SD-1	308.6	9.7	19.3	10	169	58
SD-2	336.0	10.5	20.6	12	173	55
SD-3	367.3	11.4	21.0	12	178	47
SD-4	389.5	11.7	21.6	12	182	39
SD-5	403.6	12.1	22.5	12	190	32

**Table 4:** Compressive and flexural strength decline rate.

Group	Compressive Strength				Flexural Strength			
	10 mm		20 mm		10 mm		20 mm	
	7-Days	28-Days	7-Days	28-Days	7-Days	28-Days	7-Days	28-Days
SD-1	16.36%	16.83%	40.00%	36.37%	14.41%	15.43%	7.63%	28.40%
SD-2	22.31%	21.81%	41.94%	36.84%	15.32%	8.97%	17.74%	12.18%
SD-3	16.84%	23.18%	37.21%	39.28%	25.0%	13.95%	27.70%	22.67%
SD-4	18.60%	18.83%	42.32%	31.36%	27.85%	12.50%	29.11%	25.00%
SD-5	18.66%	19.92%	36.57%	33.75%	32.22%	11.46%	35.00%	20.83%

**Table 5:** Anisotropy coefficients of 3D printed ferronickel slag–GGBFS concrete.

Nozzle Diameter	Group	Anisotropic Coefficient (*I_a_*)			
		7-d Compressive Strength	28-d Compressive Strength	7-d Flexural Strength	28-d Flexural Strength
10 mm	SD-1	7.40	6.05	1.06	0.35
	SD-2	5.62	4.34	0.35	0.14
	SD-3	6.26	5.69	0.35	0.42
	SD-4	6.84	8.51	0.28	0.21
	SD-5	5.23	4.20	0.28	0.57
20 mm	SD-1	6.58	2.04	0.49	0.28
	SD-2	5.52	2.26	0.57	0.49
	SD-3	6.65	4.60	0.35	0.92
	SD-4	5.11	5.59	0.42	0.00
	SD-5	6.39	4.82	0.35	0.28

## Data Availability

The original contributions presented in this study are included in the article. Further inquiries can be directed to the corresponding author.
